# Postpartum Respiratory Distress Due to Hypertension-Related Pulmonary Edema

**DOI:** 10.7759/cureus.18179

**Published:** 2021-09-21

**Authors:** Johnny S Randhawa, Hamza Ashraf, John Paul Colombo, Paul Kudla

**Affiliations:** 1 Internal Medicine, Ascension St. John Hospital, Detroit, USA; 2 Medicine, Saint Peter’s University Hospital, New Brunswick, USA; 3 Medical Research, Saint Peter's University Hospital, New Brunswick, USA; 4 Medical Research, St. George's University School of Medicine, True Blue, GRD

**Keywords:** hypertension related respiratory distress, postpartum hypertension, postpartum complication, postpartum respiratory distress, postpartum pulmonary edema

## Abstract

We present the case of a 35-year-old female who presented to the emergency department (ED) shortly after undergoing a cesarean section with dyspnea. Her vitals on admission revealed hypertension, tachypnea, bradycardia, and suboptimal oxygen saturation. Physical examination was remarkable for crackles in the lower lung fields. Laboratory results revealed elevated lactate dehydrogenase (LDH), pro-B-type natriuretic peptide (pro-BNP), and D-dimer levels. A CT angiogram showed no pulmonary emboli, and an echocardiogram revealed a normal ejection fraction and no diastolic dysfunction. A chest X-ray was significant for pulmonary edema and vascular congestion. The patient was diagnosed with respiratory distress due to pulmonary edema that was secondary to hypertension. This unusual case report seeks to highlight the idea that elevated blood pressure in postpartum women should warrant careful monitoring, as its consequential manifestations may be lethal. Additionally, pulmonary edema secondary to hypertension should be considered as a differential in either postpartum or peripartum women who present with respiratory symptoms and elevated blood pressure.

## Introduction

The complex nature of the adaptations that occur during pregnancy renders women susceptible to a variety of pathologies. Common symptomatic manifestations of pregnancy include urinary frequency, fatigue, poor sleep, nausea, vomiting, back pain, and pelvic pain [1,2]. Additionally, 5-10% of pregnancies are complicated by disorders of hypertension ranging from chronic hypertension and gestational hypertension to preeclampsia [3]. Delivery of the fetus and the placenta typically triggers the resolution of pregnancy symptoms and recovery to the nonpregnant state of various organs [4]. On the contrary, blood pressure ends up peaking three to six days after delivery, regardless of whether the pregnancy was complicated by a disorder of hypertension [5]. In this report, we present a case of pulmonary edema secondary to postpartum hypertension in a patient whose pregnancy was not complicated by a disorder of hypertension.

## Case presentation

**Figure 1:**
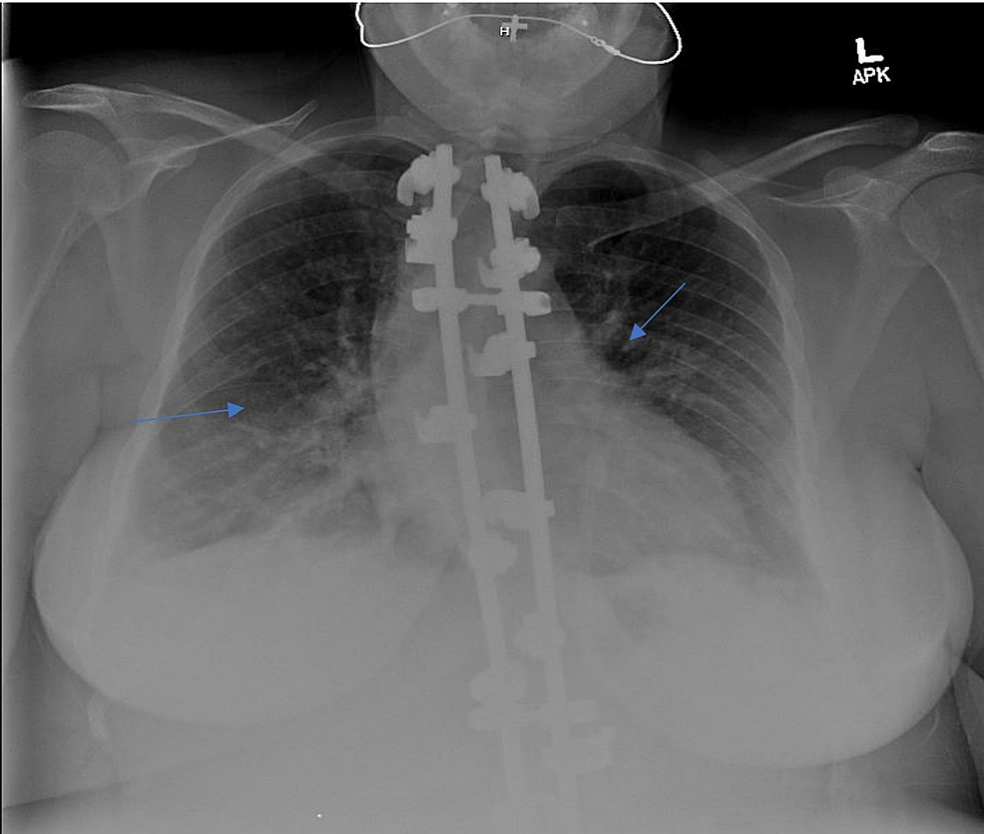
Chest X-ray showing pulmonary edema with bilateral vascular congestion with mild interstitial thickening (arrows)

A 35-year-old Caucasian female with a past medical history of three cesarean sections presented to the emergency department (ED) one week after undergoing an uncomplicated cesarean delivery with worsening shortness of breath. The cesarean delivery in question had been performed at 33 weeks of gestation due to preterm labor and preterm premature rupture of membranes. The pregnancy had been complicated by gestational diabetes, which had been well controlled with diet, exercise, and a daily dose of metformin 500 mg. There had been no immediate post-surgical complications. Three days after discharge, the patient had begun to experience worsening difficulty in breathing and reported to the ED. The shortness of breath had initially occurred only after exertion; however, it had progressed on the last day to occur even at rest. Furthermore, the patient endorsed respiratory distress when lying flat as well as waking up in the middle of the night to catch her breath. The patient stated that the root cause of her decision to seek medical care was her worsening respiratory symptoms coupled with an at-home pulse oximeter reading of 77% oxygen saturation on room air. In the ED, she was found to have an oxygen saturation of 89% on room air and was started on 4 L of oxygen therapy via nasal cannula. Consequently, her saturation improved to 93%. Vitals were significant for an elevated blood pressure of 163/86 mmHg, a heart rate of 52 beats per minute (bpm), and a respiratory rate of 28 breaths per minute. The patient was afebrile with a temperature of 97.7 °F. Physical examination was remarkable for fine bibasilar crackles bilaterally. Laboratory values showed leukocytosis (12.66 K/mcL) with a neutrophil predominance (78.4%); however, this was attributed to recent steroid use. There was no evidence of anemia, polycythemia, or any electrolyte abnormalities. A urinalysis was also unremarkable. The patient’s lactate dehydrogenase (LDH) was elevated to 309 IUnits/L, her D-dimer was elevated to 1170 ug/L UNIT, and her pro-B-type natriuretic peptide (pro-BNP) level was elevated to 693 pg/mL. Troponins were negative. A chest X-ray revealed evidence of pulmonary edema and bilateral vascular congestion with interstitial thickening (Figure 1). A CT angiogram was performed due to the patient’s elevated D-dimer levels; however, it revealed no evidence of the presence of a pulmonary embolism. A 2D echocardiogram revealed an ejection fraction of 65-70%, a moderately dilated inferior vena cava (IVC), and mild mitral regurgitation without any evidence of diastolic dysfunction. The patient was ultimately diagnosed with acute respiratory distress due to pulmonary edema that was secondary to postpartum hypertension.

After admission to the floor, the patient was started on Lasix 20 mg IV twice a day and nifedipine 60 mg daily, which led to an improvement in her symptoms and in her blood pressure. The patient was discharged two days after hospitalization with medications including nifedipine 30 mg daily and Lasix 20 mg daily. Upon discharge, the patient’s vitals were stable with a blood pressure of 119/62 mmHg, a heart rate of 65 bpm, and oxygen saturation of 95% on room air. The patient was advised to follow up with her primary care physician and obstetrician and gynecologist as an outpatient.

## Discussion

Hypertension affects approximately 7-10% of pregnancies and may persist in the postpartum period [3,6]. Hypertension in the peripartum period is defined as a systolic blood pressure of 140 mmHg or greater and/or diastolic blood pressure of 90 mmHg or greater on two or more occasions at least four hours apart [6,7]. It is a serious precursor for complications in the mother, including but not limited to a stroke, eclampsia, HELLP (Hemolysis, Elevated Liver enzymes, and Low Platelet count) syndrome, and pulmonary edema [8]. Hence, hypertension in the postpartum period requires a thorough workup as the different complications may overlap in terms of presentation and may lead to lethal sequelae. Reaching a correct diagnosis is of primary importance as it will ultimately drive a physician’s management approach [6,7,8].

The pathophysiology of maternal hypertension is related to a host of factors affecting the functionality of the cardiovascular and renal systems to meet the metabolic needs of both the mother and fetus [9]. Firstly, maternal blood volume and cardiac output increase by about 40-50%, whereas systemic vascular resistance and mean arterial blood pressure tend to decrease [9,10]. There is a marked increase in renal plasma flow and glomerular filtration rate by about 30-40% [9]. Aside from the physiological changes, there are additional changes to the systemic and renal vasculature. Using their own research along with compiled evidence, Granger et al. have come up with a potential mechanism for hypertension in pregnancy [9]. The inciting event is said to be due to abnormal cytotrophoblast invasion of spiral arterioles leading to uteroplacental ischemia. This, in turn, leads to widespread activation of the maternal vascular endothelium, resulting in the increased expression of endothelin (ET) and thromboxane (TBX), a decreased expression of prostacyclin (PGI2) and nitric oxide (NO), and increased vascular sensitivity to angiotensin II (Ang II) [9,10,11]. There are also decreases in angiogenic factors such as vascular endothelial growth factor (VEGF) and placental growth factor (PlGF) and increased concentrations of their antagonist soluble FMS-like tyrosine kinase 1 (sFlt1) [12]. The increased sFlt1/PlGF ratio hinders the binding of proangiogenic factors to their endothelial receptors, reducing NO synthesis, which is a crucial factor in vasodilation and vascular remodeling, which could otherwise possibly ameliorate the uteroplacental ischemia [12]. Together, these factors greatly reduce renal pressure natriuresis and increase systemic vascular resistance leading to a chronic hypertensive state [9].

Early-onset hypertension and/or preeclampsia occurs before 34 weeks of gestation and is attributed to syncytiotrophoblast stress leading to poor placentation, whereas late-onset hypertension and/or preeclampsia occurs at 34 weeks onward and is thought to be secondary to the placenta outgrowing its circulation [12,13]. Up to 27.5% of women develop de novo hypertension in the postpartum period, which is thought to be caused by the mobilization of fluid from the interstitial into intravascular space, administration of fluids, and vasoactive agents [12]. This shift of fluids increases the stroke volume and cardiac output up to 80%, which is then followed by compensatory vasodilation and diuresis, which eventually stabilizes the rise in blood pressure [12].

Dyspnea in a postpartum female may occur due to a variety of underlying etiologies. The differential diagnoses to consider for postpartum dyspnea are listed in Table 1. Our case presentation was very similar to a case report by Dunne and Meriano, in that both of our patients presented with acute-onset postpartum dyspnea; however, we were unable to establish a formal diagnosis from the aforementioned differentials by ruling out each diagnosis one by one [14]. Herein, we discuss how we ruled out each of these diagnoses for our patient.

**Table 1:** Differential diagnoses of acute dyspnea in the postpartum patient stratified by pulmonary edema vs. non-pulmonary edema causes

Without pulmonary edema	With pulmonary edema	
	Cardiogenic	Non-cardiogenic
Pulmonary embolism	Peripartum cardiomyopathy	Iatrogenic fluid overload
Amniotic fluid embolism	Preeclampsia-related heart failure	Thyroid disease
Pneumonia	Underlying cardiac disease (e.g., valvulopathy)	Tocolytic therapy
Foreign body aspiration	Myocardial ischemia	Medication-related sepsis
Psychogenic dyspnea	Sepsis with poor cardiac output	Acute respiratory distress syndrome

Our patient, who presented with an acute episode of respiratory distress, was initially thought to have peripartum cardiomyopathy based on her recent c-section, the pattern of dyspnea, orthopnea, pitting edema, and chest X-ray findings significant for pulmonary edema. In adults, peripartum cardiomyopathy has a prevalence of 0.2-0.4%, accounting for 11% of maternal deaths, and can occur up to one month after pregnancy [15,16]. Studies have also shown that there is a strong association between postpartum cardiomyopathy and gestational hypertension [17]. Additionally, an elevated BNP level of 683 in our patient further misled us to suspect a cardiovascular source as studies have shown that an increase in left ventricular mass was associated with elevated ANP and BNP levels, suggesting a left ventricular dysfunction [18]. This could be possibly explained due to cardiac remodeling that the heart undergoes during pregnancy from the pressure overloaded state. This is to reduce wall stress associated with increased afterload [18]. The echocardiogram results ultimately revealed an ejection fraction of 65-70% with a normal left ventricular diastolic function, and the presumptive diagnosis of peripartum cardiomyopathy was ultimately ruled out.

Another diagnosis that should be ruled out is a pulmonary embolism as the risk for postpartum venous thromboembolism (VTE) is increased and may persist for up to 12 weeks [19]. The risk is estimated to be around 0.05% and is highest in the first six weeks postpartum [19]. Although our patient denied any symptoms of chest pain, she did present with other commonly seen symptoms of dyspnea, peripheral edema, and showed an elevated D-dimer level of 1,170 ngFEU/mL on her first admission. A CT angiogram was performed, which was negative for any pulmonary embolism, which effectively ruled out this diagnosis.

Although rare, a diagnosis of postpartum preeclampsia was also considered as part of the initial assessment and plan. Acute pulmonary edema occurs in 0.08-0.5% of women during pregnancy and the postpartum period and is commonly caused by preeclampsia [20,21]. The pathophysiology of pulmonary edema related to preeclampsia is thought to be multifactorial [14]. The predisposition of pulmonary edema in postpartum pregnancy (38%) involves a complex interplay between increased intravascular hydrostatic pressures, increased permeability of the vascular system, and decreased intravascular colloid osmotic pressures, especially in preeclampsia, leading to fluid extravasation into the pulmonary interstitium [14,21,22]. However, not all cases of pulmonary edema are due to this phenomenon [14,21]. Our patient presented with postpartum hypertension with flash pulmonary edema and did not meet the criterion for preeclampsia, as she did not have significant proteinuria (30 mg/dL) on urinalysis.

The rest of the aforementioned diagnoses were ruled out with high certainty based on the patient’s presentation itself. Amniotic fluid embolism is a rare but life-threatening complication that occurs usually during labor and delivery and presents with acute severe dyspnea, hypoxemia, and hypotension followed by cardiac arrest within minutes [23]. This was effectively ruled out due to the presentation of dyspnea a week following delivery, the absence of hypotension, and left ventricular dysfunction on echocardiography. Tocolytics are β-mimetic agents that have been widely used in the treatment of premature labor, which could induce pulmonary edema in 0.5-5% of pregnancies. They stimulate β2-adrenergic receptors, which increase pulse rate and cardiac output and cause peripheral vasodilation also leading to hemodilution marked by a drop in the hemoglobin, hematocrit, and albumin. During delivery, the uterine contractions cause autotransfusion, and increased venous tone and blood pressure lead to pulmonary edema in the postpartum period [23]. Our patient, despite being of premature gestational age, did not receive tocolytic therapy. Lastly, we had to rule out iatrogenic fluid overload as a cause of our patient’s pulmonary edema. According to a study by Sciscione et al., which examined 10 years of pregnancy data for pulmonary edema, iatrogenic fluid overload, tocolytic use, and cardiac disease were the most common causes of postpartum pulmonary edema [24]. Those patients with iatrogenic fluid overload as the etiology of their pulmonary edema had a mean fluid balance of 6,022 ± 3,340 mL [24] However, our patient presented a week later; moreover, a careful look at her intake and output revealed that her net fluid balance was significantly less than that reported in the above-mentioned study.

## Conclusions

The presentation of acute dyspnea in postpartum patients is uncommon; however, it necessitates a full workup due to the potential for high-risk disease that carries a high mortality rate. Thus, when a postpartum patient with symptoms of dyspnea is admitted, it is imperative that close monitoring for all disorders including peripartum cardiomyopathy, postpartum preeclampsia, pulmonary embolism, and other causes of postpartum flash pulmonary edema be performed. A thorough examination involving a stat echocardiogram, chest X-ray, EKG, urinalysis, and repeat blood pressures should be conducted as an initial workup. The treatment for postpartum pulmonary edema is largely supportive, using respiratory support as needed, diuretics, and blood pressure control.
